# A neural network approach to glomerular filtration rate estimation: a single-centre retrospective audit

**DOI:** 10.1097/MNM.0000000000001982

**Published:** 2025-04-07

**Authors:** Jack A. Johnson, Richard Meades, Nathan J. Dickinson

**Affiliations:** a Department of Oncology, University of Oxford, Oxford; b Nuclear Medicine Department, The Royal Free Hospital London NHS Foundation Trust, London; c Nuclear Medicine Department, University Hospitals of Leicester NHS Trust, Leicester, UK

**Keywords:** eGFR, GFR, glomerular filtration rate, machine learning, neural network

## Abstract

**Objectives:**

The 2009 Chronic Kidney Disease Epidemiology Collaboration (CKD-EPI) equation without race correction factor is frequently used for an estimate of glomerular filtration rate (eGFR) and to support a single-sample GFR regime. This study examines whether neural networks offer a potential means to improve the accuracy of GFR estimates using the same initial inputs as eGFR.

**Methods:**

An audit of 865 adult GFR examinations and serum creatinine measurements between January 2010 and 2024 was undertaken. Patient sex, age, creatinine, and measured GFR were used to train a neural network (NN) model with an 80 : 20 train-test split, with test set root mean square error (RMSE), accuracy, median bias, and sensitivity calculated and compared against the 2009 CKD-EPI equation eGFR.

**Results:**

NN GFR showed an improved performance against the 2009 CKD-EPI equation in RMSE: 12.0 vs. 16.6 mL/min/1.73 m^2^ (*P* < 0.001), median bias: −2.50 vs. 7.86 mL/min/1.73 m^2^ (*P* < 0.001) and accuracy: 94.2 vs. 83.2% (*P* < 0.001). Both NN GFR and the eGFR equation had poor sensitivity across the British Nuclear Medicine Society single-sample ranges of 25–50, 50–70, 70–100, and >100 mL/min/1.73 m^2^: 57.9 vs. 57.9%, 50.0 vs. 26.9%, 84.4 vs. 54.2%, 10.0 vs. 70.0%.

**Conclusion:**

This study has suggested that locally trained NNs can offer a potential avenue to improve GFR predictions, even on small and diverse datasets.

**Advances in knowledge:**

Although the model is not sufficiently sensitive to predict the optimum time-sample point for a single-sample regime, this work can serve as a proof of concept for UK-specific NN GFR models.

## Introduction

Glomerular filtration rate (GFR) is frequently used as a measure of patient kidney function, representing the volume of plasma presented to the nephrons per unit time during urine formation. Accurate assessment of GFR is important to kidney disease diagnosis, as well as monitoring disease severity and progression. GFR is often measured using plasma sampling, where ^99m^Tc-diethylenetriamine pentaacetate is administered intravenously and subsequently cleared by the kidneys. The GFR value can then be calculated through the quantification of plasma and standard activity using a gamma counter [1]. The 2004 British Nuclear Medicine Society (BNMS) Clinical Guidelines recommended taking multiple plasma samples to calculate GFR using a slope-intercept technique [2], often requiring up to four patient blood samples. Aside from nuclear medicine techniques, GFR can be estimated (eGFR) through patient demographic and blood biomarker values. In the UK, as in most European countries, the 2009 Chronic Kidney Disease Epidemiology Collaboration (CKD-EPI) equation without race correction factor is frequently used, taking patient age, sex, and serum creatinine to produce eGFR (Equation 1), as per the National Institute for Health and Care Excellence 2021 guidelines [3,4].


$$\begin{array}{lll} GFR & =141\times min{\left(\frac{Scr}{\kappa },1\right)}^{\alpha }\times max{\left(\frac{Scr}{\kappa },1\right)}^{-1.209}\; & \times 0.993{}^{Age}\times \left(1.018\;\;if\;female\right), \end{array}$$ (1)

where, Scr = serum creatinine (mg/dL),

*κ* = 0.7 for females and 0.9 for males,

*α* = −0.329 for females and − 0.411 for males.

Whilst eGFR is often used as a quick approximation of patient GFR without the need for full nuclear medicine investigation, accurate eGFR could also assist with the examination itself. In 2018 BNMS recommended the use of a single-sample GFR technique [5]. This could result in an improved patient experience and resource savings for nuclear medicine departments by collecting fewer blood samples. However, in a single-sample GFR exam, the optimum time-point to take a plasma sample is itself dependent on GFR. This leads to a challenging scenario where one needs to know a patient’s GFR in advance of taking a measurement. Recent work by Bonney *et al*. [6] investigated the use of the 2009 CKD-EPI eGFR equation without race correction factor in predicting the optimum sample time-point for a single-sample GFR. They found that the 2009 CKD-EPI eGFR equation had a high root mean square error (RMSE) on the true, measured GFR (mGFR) of 19.3 mL/min/1.73 m^2^ and a low sensitivity for predicting the optimum time-point, and was therefore not sufficiently accurate to predict the optimum sample time-point [6].

Neural networks (NNs) trained on local patient data offer the potential to improve the accuracy of eGFR using the same inputs as the 2009 CKD-EPI equation, because of their ability to model complex non-linear relationships between variables, potentially leading to improved modelling sensitivity. Whilst there is limited work on using NNs to improve eGFR accuracy in the literature, Li *et al*. [7], Jiang *et al*. [8], and Liu *et al*. [9] used datasets comprising patients in China and Wang *et al*. [10], applied a NN model to a dataset composed of patients in the USA. The work of Jiang *et al*. [8], and Liu *et al*. [9] did see significant accuracy improvements, but Liu *et al*. noted that further work was required on more diverse datasets. On the other hand, the work of Wang *et al*. [10] utilised a larger and much more diverse dataset but implemented a race correction factor for a significant portion of their work. Our work therefore focuses on testing an NN model on a single-centre using a more diverse dataset. In the UK, Leicester is a diverse city with 43.4 and 7.8% of inhabitants identifying as Asian or Asian British and Black or Black British, respectively [11], and thus represents an opportunity to test a NN model on a diverse population without implementing an ethnicity correction factor.

## Methods

### Dataset

A retrospective audit of 1308 adult GFR exams between January 2010 and 2024 using the multisample slope-intercept method (as per the 2004 BNMS guidelines) and corresponding serum creatinine eGFR measurements was undertaken at the University Hospitals of Leicester NHS Trust. GFR studies were excluded if they did not have an associated serum creatinine measurement within 28 days of the GFR exam, in line with previous work in the field [12], or if the plasma volume of dilution was outside the expected range for the patient, as advised in the 2018 BNMS guidelines (*n* = 443 total) [5]. The resulting 865 GFR studies were then randomly divided into an 80 : 20 train-test split (Table 1) using scikit-learn’s train-test split feature [13].

**Table 1:** Training and test set characteristics

Characteristics	Training set (*n* = 692)	Test set (*n* = 173)
Mean age (years)	48.7 ± 14.6	49.4 ± 15.5
Males, *n*	342 (49.4%)	97 (56.1%)
Mean serum creatinine (mg/dL)	0.949 ± 0.443	0.955 ± 0.347
Mean mGFR (mL/min/1.73 m^2^)	81.3 ± 22.6	79.5 ± 21.9
mGFR < 25 mL/min/1.73 m^2^	17 (2.46%)	2 (11.6%)
mGFR 25–50 mL/min/1.73 m^2^	61 (8.82%)	19 (11.0%)
mGFR 50–70 mL/min/1.73 m^2^	72 (10.4%)	26 (15.0%)
mGFR 70–100 mL/min/1.73 m^2^	418 (60.4%)	96 (55.5%)
mGFR ≥ 100 mL/min/1.73 m^2^	124 (17.9%)	30 (17.3%)

mGFR, measured GFR.

### Feature engineering and selection

From the initial data collected (age, sex, serum creatinine, and mGFR for each datapoint), feature engineering and feature selection using the searching for an uncorrelated list of variables (SULOV) method, followed by recursive XGBoost method, was undertaken using the FeatureWiz Python library [14]. The resulting input features were then transformed using MinMax scaling. This normalises the features, so each value now lies on a scale between 0 and 1, hence the minimum and maximum values of an input feature are 0 and 1, respectively. Having input features on a comparable scale avoids the problem of having larger scale features dominating the training process, hence making inputs more appropriate for application in NN modelling.

### Model development and hyperparameter tuning

An in-house artificial NN model was created with Python v3.11.5 (Python Software Foundation, Wilmington, Delaware, USA) using a Jupyter Notebook v6.5.4 (Project Jupyter) [15,16]. It was run on 11th Gen Intel(R) Core i5-1135G7 with 2.40 GHz processors and 16.0 GB RAM. Hyperparameter tuning was undertaken by grid search using the Keras Tuner Python library [17]. The number of neurons in the hidden layers (ranging between 1 and 64) for each of the two layers and the model learning rate (ranging between 0.0001 and 0.01) were chosen based on training set RMSE. These hyperparameters, along with the selected features, made up the final model architecture which was applied to the unseen test set.

### Model evaluation and statistical analysis

The test set performance of our model (henceforth referred to as NN GFR) and the 2009 CKD-EPI equation eGFR (henceforth referred to as eGFR) was assessed by RMSE, accuracy (defined in previous literature [8,18] as the percentage of eGFR or NN GFR values within 30% of their corresponding mGFR values), median bias (the median difference between NN GFR or eGFR and mGFR), mean bias (the mean difference between NN GFR or eGFR and mGFR) and precision (the interquartile range for this difference). 95% confidence intervals (CIs) for the results were calculated using the bootstrap method (1000 bootstraps). *F*-test, exact binomial test, Wilcoxon signed-rank test, Student’s *t*-test, and permutation test (1000 permutations) were used to assess the respective significance of the difference in RMSE, accuracy, median bias, mean bias, and precision between the two models.

**Fig. 1:**
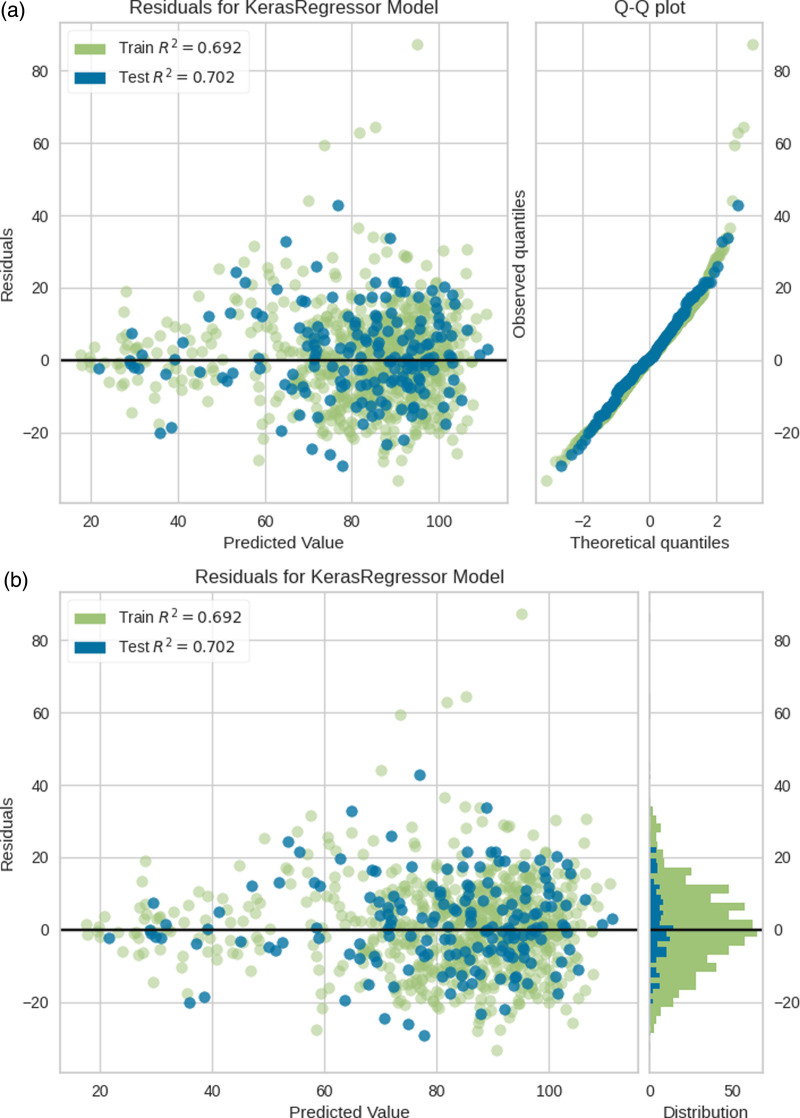
Plots of residuals and NN GFR, along with *Q*–*Q* plots (a) and histograms (b) for the training and test set. The *R*^2^ scores for the training and test set were 0.692 and 0.702, respectively. GFR, glomerular filtration rate; NN, neural network; Q-Q, quantile-quantile.

The differences between NN GFR and mGFR were visualised by producing plots of residuals against mGFR for both the training and test set. Quantile–quantile plots (*Q*–*Q* plots) and histograms, as well as *R*^2^ scores for the training and test datasets, were created to compare the distribution of residuals to a Gaussian distribution (Fig. 1a and b).

The sensitivity of NN GFR and eGFR was also assessed in a similar fashion to Bonney *et al*. using the ranges proposed by BNMS in 2018. In these guidelines, the BNMS recommended time-point for a single-sample GFR is selected based upon patient eGFR, as per Table 2 [5].

**Table 2:** British Nuclear Medicine Society recommended sampling times for single-sample glomerular filtration rate measurements for patient estimated glomerular filtration rate ranges

Patient eGFR	BNMS recommended post-injection sample time (h)
25–50 mL/min/1.73 m^2^	6
50–70 mL/min/1.73 m^2^	4
70–100 mL/min/1.73 m^2^	3
>100 mL/min/1.73 m^2^	2

BNMS, British Nuclear Medicine Society; eGFR, estimated glomerular filtration rate.

To be effective in a single-sample regime, the model must have a high sensitivity, where the number of true positives is maximised and the number of false negatives minimised, as per Equation 2.


$$\begin{array}{l} Sensitivity=\frac{True\;Positives}{\left(True\;Positives\;+\;False\;Negatives\right)}. \end{array}$$ (2)

The specificity of the model (Equation 3) was also assessed and compared with eGFR using the 2018 BNMS ranges.


$$\begin{array}{l} Specificity=\frac{True\;Negatives}{(True\;Negatives\;+\;False\;Positives)}. \end{array}$$ (3)

The following categories are defined as such: true positive: NN GFR or eGFR correctly predicts the mGFR value within a range. True negative: NN GFR or eGFR correctly predicts the mGFR value outside of a range. False positive: NN GFR or eGFR incorrectly predicts an mGFR value within a range. False negative: NN GFR or eGFR incorrectly predicts an mGFR value outside of a range.

## Results

### Feature selection and engineering

The initial feature engineering resulted in the creation of 15 additional interaction features, resulting in 18 features in total. From these, five input features were selected by the SULOV method: age multiplied by serum creatinine, age divided by serum creatinine, sex subtracted from age, sex multiplied by creatinine, and sex multiplied by age.

### Model architecture

From hyperparameter tuning, the final NN GFR model architecture consisted of five neurons in the input layer (for the aforementioned selected features), two hidden layers of 36 and 41 neurons using the leaky rectified linear unit (*α* = 0.01) activation function, and an output layer with one neuron using a linear activation function for the model’s GFR prediction. The NN GFR model used a learning rate of 0.01, an Adam optimiser, an RMSE loss function, a batch size of 16 and was trained for 100 epochs, with early stopping implemented if the model did not see an improvement in the validation set RMSE after 10 epochs [19].

### Model predictions and analysis

**Fig. 2:**
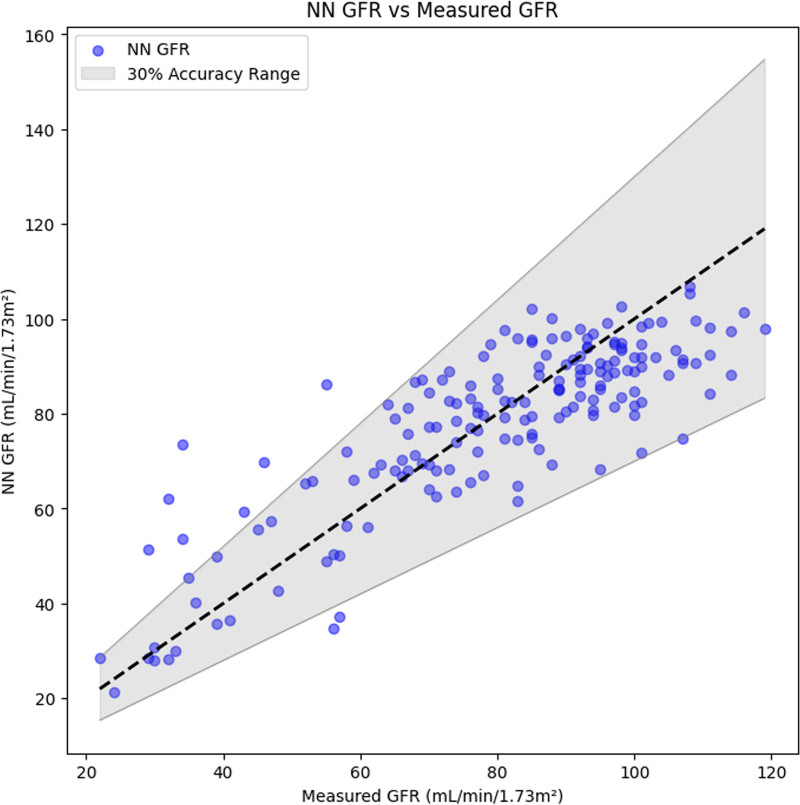
NN GFR vs. mGFR for the test set (*n* = 173). NN GFR had a smaller RMSE of 12.0 mL/min/1.73 m^2^ and a higher accuracy of 94.2%. GFR, glomerular filtration rate; mGFR, measured GFR; NN, neural network; RMSE, root mean square error.

**Fig. 3:**
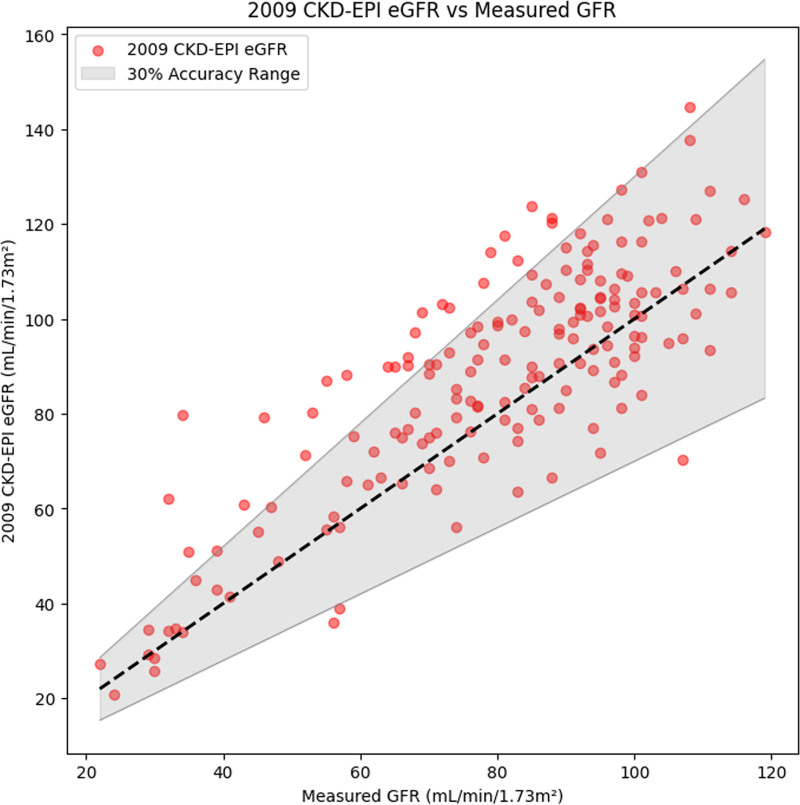
eGFR vs. mGFR for the test set (*n* = 173). The eGFR had a RMSE of 16.6 mL/min/1.73 m^2^ and an accuracy of 83.2%. CKD-EPI, Chronic Kidney Disease Epidemiology Collaboration; eGFR, estimated glomerular filtration rate; mGFR, measured glomerular filtration rate; NN, neural network; RMSE, root mean square error.

**Fig. 4:**
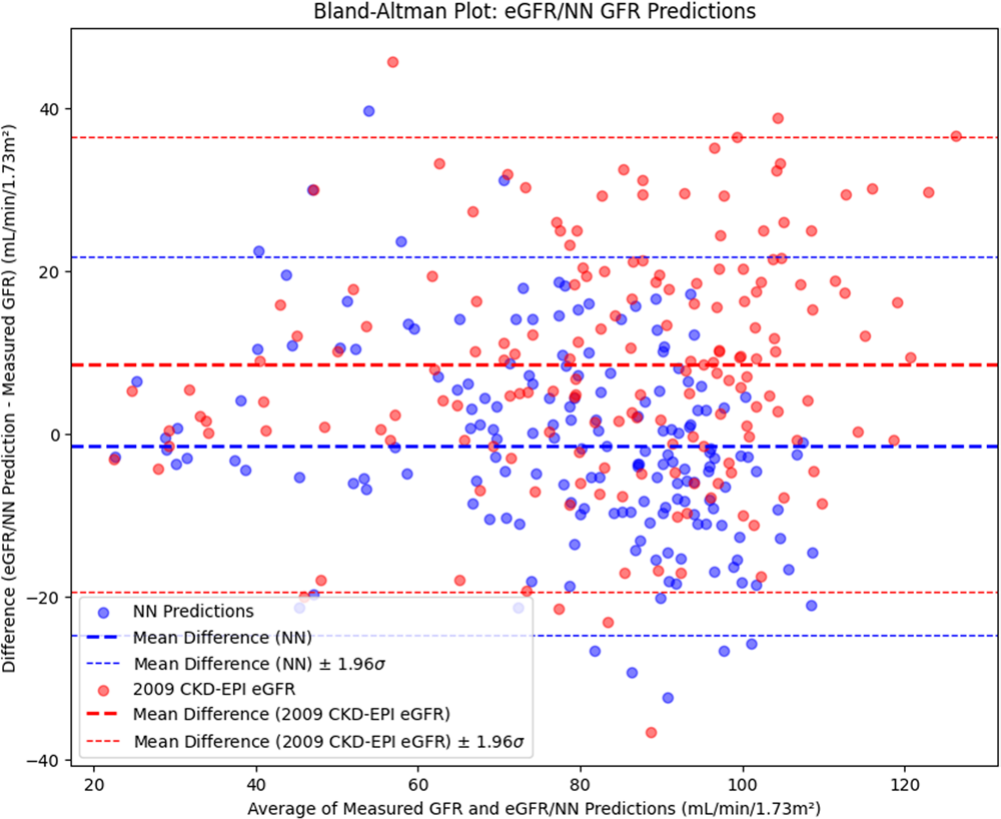
Bland–Altman NN GFR/eGFR vs. measured GFR. The NN GFR and eGFR had mean biases of –1.50 and 8.49 mL/min/1.73 m^2^, respectively. CKD-EPI, Chronic Kidney Disease Epidemiology Collaboration; eGFR, estimated glomerular filtration rate; GFR, glomerular filtration rate; NN, neural network.

The NN GFR demonstrated superior performance compared with eGFR in RMSE (*P* < 0.001), median bias (*P* < 0.001), accuracy (*P* < 0.001) (NN GFR shown in Fig. 2 and eGFR shown in Fig. 3), and mean bias (*P* < 0.001) (Fig. 4), but with a comparable precision value (*P* = 0.051). Full results and 95% CIs for both NN GFR and eGFR are noted in Table 3.

**Table 3:** Estimated glomerular filtration rate and neural network glomerular filtration rate root mean square error, accuracy, median bias, mean bias and precision with 95% confidence intervals for the test set

	RMSE (95% CI) (mL/min/1.73 m^2^)	Accuracy (95% CI) (%)	Median bias (95% CI) (mL/min/1.73 m^2^)	Mean bias (95% CI) (mL/min/1.73 m^2^)	Precision (95% CI) (mL/min/1.73 m^2^)
NN GFR	12.0 (10.6–13.3)	94.2 (90.8–97.7)	−2.50 (−3.95 to −0.499)	−1.50 (−3.35 to 0.261)	15.0 (11.9–17.6)
eGFR	16.6 (14.8–18.1)	83.2 (77.5–88.4)	7.86 (4.81–10.1)	8.49 (6.22–10.5)	19.3 (16.3–21.5)

CI, confidence interval; eGFR, estimated glomerular filtration rate; GFR, glomerular filtration rate; NN, neural network; RMSE, root mean square error.

**Fig. 5:**
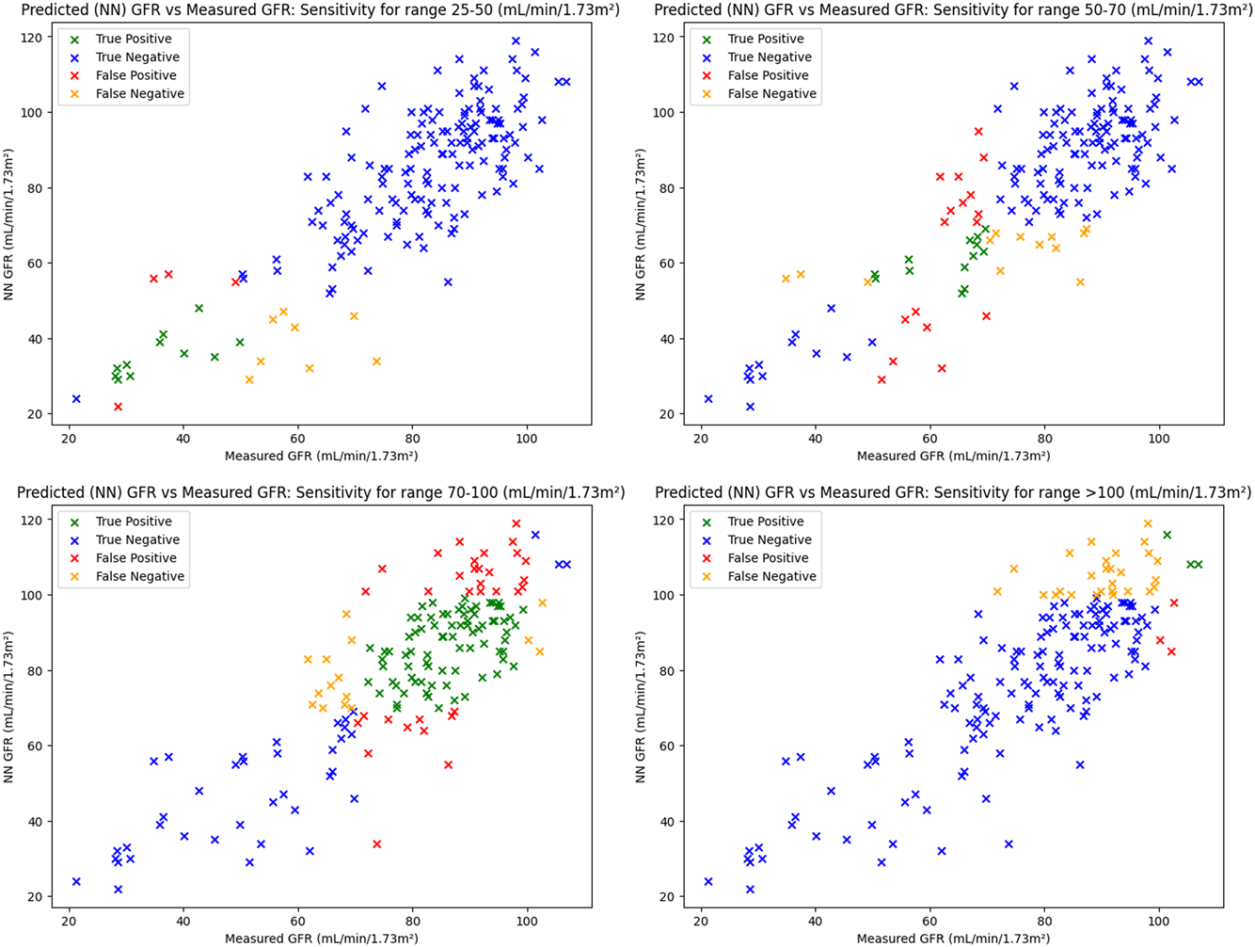
(a–d) Plots of NN GFR vs. mGFR for the BNMS ranges 25–50, 50–70, 70–100, and more than 100 mL/min/1.73 m^2^, with respective sensitivities of 57.9, 50.0, 84.4, and 10.0%. BNMS, British Nuclear Medicine Society; GFR, glomerular filtration rate; mGFR, measured glomerular filtration rate; NN, neural network.

**Fig. 6:**
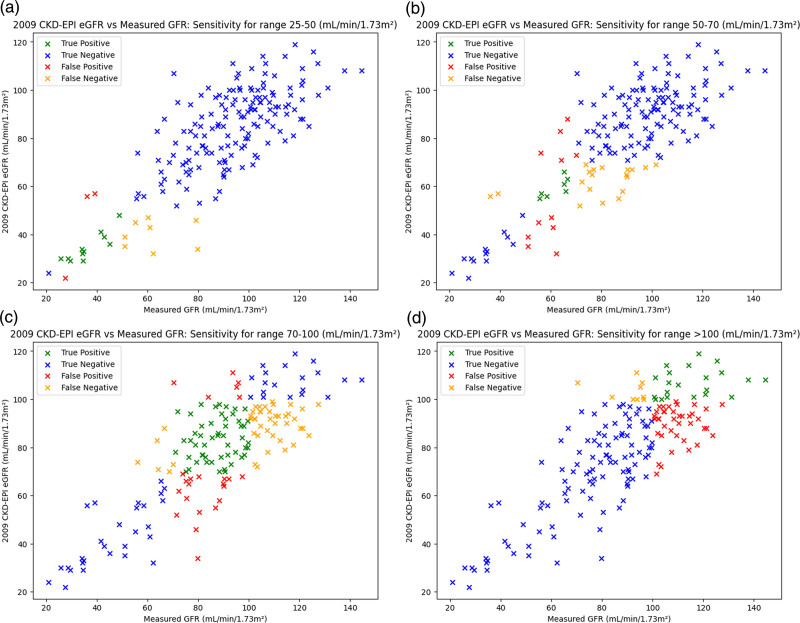
(a–d) Plots of eGFR vs. mGFR for the BNMS ranges 25–50, 50–70, 70–100, and more than100 mL/min/1.73 m^2^, with respective sensitivities of 57.9, 26.9, 54.2, and 70.0%. BNMS, British Nuclear Medicine Society; CKD-EPI, Chronic Kidney Disease Epidemiology Collaboration; eGFR, estimated glomerular filtration rate; GFR, glomerular filtration rate; mGFR, measured glomerular filtration rate.

However, eGFR and NN GFR demonstrated inconsistent sensitivity performance across the ranges proposed in the BNMS guidelines. NN GFR and eGFR sensitivity for the ranges proposed by BNMS are noted in Table 4, Figs. 5a–d, and 6a–d, respectively. The specificity for NN GFR and eGFR for the BNMS ranges is also detailed in Table 5.

**Table 4:** Sensitivities for neural network glomerular filtration rate and estimated glomerular filtration rate across the single-sampling ranges proposed by British Nuclear Medicine Society

Sensitivity	25–50 mL/min/1.73 m^2^	50–70 mL/min/1.73 m^2^	70–100 mL/min/1.73 m^2^	>100 mL/min/1.73 m^2^
NN GFR	57.9%	50.0%	84.4%	10.0%
eGFR	57.9%	26.9%	54.2%	70.0%

eGFR, estimated glomerular filtration rate; GFR, glomerular filtration rate; NN, neural network.

**Table 5:** Specificities for neural network glomerular filtration rate and estimated glomerular filtration rate across the single-sampling ranges proposed by British Nuclear Medicine Society

Specificity	25–50 mL/min/1.73 m^2^	50–70 mL/min/1.73 m^2^	70–100 mL/min/1.73 m^2^	>100 mL/min/1.73 m^2^
NN GFR	97.4%	88.0%	54.2%	97.9%
eGFR	98.1%	92.2%	66.2%	72.7%

eGFR, estimated glomerular filtration rate; GFR, glomerular filtration rate; NN, neural network.

## Discussion

Considering the small sample size, limited number of input features, and relatively narrow hyperparameter tuning, the NN GFR model demonstrated promising performance on the test dataset. The overall accuracy of 94.2% and median bias of −2.50 mL/min/1.73 m^2^, as well as the model’s performance in the 70–100 mL/min/1.73 m^2^ range, suggests that NN models could accurately predict GFR values given sufficiently large training sets. In the 70–100 mL/min/1.73 m^2^ range, where the majority of test set GFR samples were located, the NN GFR model had a sensitivity of 84.4%, but its lower sensitivity across more extreme GFR values, as indicated by the 10.0% sensitivity in the greater than 100 mL/min/1.73 m^2^ region, would limit its usage in supporting a single-sample GFR method. Similarly, at lower GFR ranges (25–50 mL/min/1.73 m^2^), the NN model had a low sensitivity performance of 57.9% and thus did not see an improvement upon eGFR for this GFR range. There are several different explanations for the poor sensitivity performance. The RMSE loss function used in training the model, whilst resistant to outliers, is perhaps slightly conservative with its GFR predictions, leading to a negative bias for NN predictions. This often pushes NN GFR predictions just below their true time sampling point range, leading to poorer sensitivity, particularly in the greater than 100 mL/min/1.73 m^2^ range. At lower GFR ranges (25–50 mL/min/1.73 m^2^), additional uncertainties in sample collection could have also led to poorer sensitivity in this range.

This work has several limitations and there are several approaches which could be taken to build upon this and improve modelling. This model was tested on a small dataset of just 865 samples, with previous papers featuring over 22,000 GFR samples. This leaves the model vulnerable to overfitting and modelling noise, and validation on an external dataset would also be required before implementing a NN GFR model clinically. The relatively limited number of GFR values in the 25–50 and greater than 100 mL/min/1.73 m^2^ ranges in the dataset (only 9.25 and 17.8%, respectively) meant that the model could be overtraining on the majority of GFR values, thus leading to the aforementioned reduction in sensitivity at more extreme GFR ranges. Therefore, sampling with more heterogeneous GFR values and incorporating larger datasets with more extreme GFR ranges would be prudent to ensure that a NN GFR model can be applicable to a wide range of patients.

The model architecture itself could also be improved upon – a more rigorous hyperparameter search, with different hidden layer architectures, batch sizes, activation functions, and loss functions could also improve the model. Limited computing resources meant that only the learning rate and the number of hidden neurons could be explored, and thus deeper hyperparameter searches could lead to improved results. More extensive feature selection could also be explored – this work focused primarily on using simple and readily available clinical data that would be commonplace across most hospital sites. Whilst making the model more accessible, this does limit the number of predictive input features that could be explored with feature engineering. A model which incorporates different features such as a patient’s weight and height, or biomarkers such as cystatin C could have greater success, albeit at the cost of collating more clinical information before being able to provide a GFR estimate. Moreover, testing other artificial intelligence approaches such as machine learning models would also be prudent, which could help to further reduce RMSE and improve model performance.

To maximise the number of samples for use in the NN model, data were collected over a large time frame, meaning that several different operators could have taken serum creatinine and GFR measurements, potentially leading to uncertainty in eGFR and mGFR values because of varying operator techniques in plasma collection, sample pipetting, and so forth. Data were also only obtained at one centre; validation work on multiple datasets would also be prudent. Finally, the NN GFR model is only compared against the 2009 CKD-EPI eGFR equation, and other eGFR equations such as the Modification of Diet in Renal Disease equation or the 2021 CKD-EPI eGFR equation have also been formulated [20,21].

Previous papers on NN applications to eGFR often conclude by revising the CKD-EPI equations with new weightings based upon their network’s results. Although further work is needed on NN and machine learning modelling on British datasets, the promising results of this study suggest that a new approach could be taken. Given the improved performance that a NN can have when trained on a small dataset, centres could take a more individualised approach when using NNs in GFR examinations. Individual hospitals could train their own models with local data, allowing NNs to make more accurate predictions based upon local demographics, instead of reverting to generalised regression equations which may not adapt well to different minority populations, although this would require data tracking and model retraining over time to prevent concept drift as demographic factors shift. However, it is important to note that rigorous testing, as well as device compliance and management in line with the 2002 Medical Devices Regulations [22], additional personnel training and validation on external datasets would be required to ensure that NNs are appropriate for clinical applications. However, this work potentially opens the door to further studies on applying NN models to GFR estimation. Locally trained models could be designed to give quick estimates for patient GFR in advance of an actual exam and could replace traditional serum creatinine equations as a method for providing eGFR.

### Conclusion

Overall, this audit can be used as a proof of concept for UK-specific NN modelling for GFR estimation. The model’s superior performance over the 2009 CKD-EPI equation eGFR in RMSE, accuracy, and median bias represents a new avenue for improvement in GFR estimation. The model performed best in the 70–100 mL/min/1.73 m^2^ region, where over half of the GFR samples were situated, demonstrating that NN techniques could be applied with reasonable success to most of the population, even with a relatively small dataset, limited feature engineering, and restricted hyperparameter tuning. Therefore, NN models have the potential in being able to provide quick, inexpensive, and reasonably reliable estimates for patient GFR without having to conduct a full nuclear medicine exam, which has applications in screening patients before an examination or assisting in a diagnosis. The model does have a few notable caveats, with poor sensitivity results at lower GFR ranges, and especially at higher GFR values. This limits the model’s utility in a single-sample regime as per the 2018 BNMS guidelines, where it is important for the GFR prediction to fall into the same optimum time-sample range as its true GFR counterpart. Further work with larger datasets, a greater number of input features, and deeper hyperparameter tuning could potentially improve model performance and make it a viable option for supporting a single-sample regime. The CKD-EPI equations are primarily based upon work done on American datasets, and NN models could provide accurate, alternative GFR estimates outside of the US, where separate equations have yet to be developed.

## Acknowledgements

This work builds upon from data presented previously at the British Nuclear Medicine Society 2024 Spring Meeting and published as abstract in Nuclear Medicine Communications (2024), 45:420–461.

### Conflicts of interest

There are no conflicts of interest.
